# Innovative Bistable Composites for Aerospace and High-Stress Applications: Integrating Soft and Hard Materials in Experimental, Modeling, and Simulation Studies

**DOI:** 10.3390/ma17174280

**Published:** 2024-08-29

**Authors:** Khalid Zouhri, Mohamed Mohamed, Anil Erol, Bert Liu, Philip Appiah-Kubi

**Affiliations:** 1 Department of Engineering Management, Systems & Technology, University of Dayton, 300 College Park, Kettering Lab 241M, Dayton, OH 45469, USA; pappiahkubi1@udayton.edu; 2 Department of Mechanical Engineering, University of Dayton, 300 College Park, Kettering Lab 241M, Dayton, OH 45469, USA; mohamedm2@udayton.edu; 3 Northrop Grumman, 2980 Fairview Park Drive, Falls Church, VA 22042, USA; anil.erol@ngc.com; 4 Structural Materials Division, University of Dayton Research Institute, 1700 South Patterson Blvd, Dayton, OH 45469, USA; bert.liu.ctr@afrl.af.mil; 5 Air Force Research Laboratory, Aerospace Systems Directorate, 1790 Loop Road N., Bldg. 490, Wright-Patterson AFB, Dayton, OH 45433, USA

**Keywords:** bistable, materials, flexible, stress, composite

## Abstract

This study explores the development and performance of bistable materials, emphasizing their potential applications in aero-vehicles and high-stress environments. By integrating soft and hard materials within a composite structure, the research demonstrates the creation of bistable composites that exhibit remarkable flexibility and rigidity. Advanced simulations using COMSOL Multiphysics and 3D-printed prototypes reveal that these materials effectively absorb and dissipate stress, maintaining structural integrity under high-pressure conditions. Compression tests highlight the ability of bistable structures to bear significant loads, distributing stress efficiently across multiple layers. The innovative proposal of combining stiff and flexible materials within a single unit cell enhances bistable behavior, offering superior energy absorption and resilience. This work underscores the promise of bistable materials in advancing materials science, providing robust solutions for aerospace, automotive, and protective gear applications and paving the way for future research in optimizing bistable structures for diverse engineering challenges.

## 1. Introduction

Bistable materials and structures are characterized by having two stable configurations, allowing them to maintain a stable equilibrium after deformation. When a deformable body is subjected to a force and the force is removed, it typically either deforms permanently or elastically returns to its original shape. Permanent deformation is often irreversible, associated with material failure or plasticity. However, bistable structures exhibit reversible permanent deformation, enabling them to switch between stable states without material degradation [1,2]. The concept of bistability, which has been understood for a long time, has recently seen a resurgence due to its potential applications in various fields. For example, in soft robotics, bistable materials provide enhanced flexibility and control. In deployable structures, they maintain their shape without continuous energy input [3,4]. Bistable designs are also valuable in actuators and reusable energy absorbers, where materials need to change shape repeatedly in response to external stimuli without sustaining permanent damage [5,6]. Recent advancements in additive manufacturing (AM) techniques, such as fused filament fabrication (FFF) and direct writing (DW), have broadened the scope for creating multistable structures [7,8]. These methods allow for the precise fabrication of one-dimensional and two-dimensional structures with tailored buckling properties, making them suitable for restorable shock absorbers. Additionally, technologies like triple jetting enable the production of more complex bistable structures, including torsional and hierarchical configurations. These designs can achieve multiple activated states depending on the direction and magnitude of the applied load. The variety of topologies proposed for bistable architectures paves the way for designing structures with specific, targeted stable configurations. This versatility allows for the development of innovative materials and devices that can adapt to various mechanical demands, driving advancements across multiple engineering and technological fields. Traditional mechatronic systems are composed of sensors, actuators, and micro-electronics that are interconnected by a complex network of wiring and cables. Each component in these systems contributes additional weight and relies on delicate electronics, making them susceptible to damage. Because of the logical construction of their periodic unit structures, lattice structures can display special characteristics not seen in nature. The capacity to produce intricately shaped lattice structures of superior quality has been made possible by the swift progress of additive manufacturing technology. The investigation of unique lattice structures with inventive and extreme features and functions has been sparked by this advancement. Lattice structures with pentamode characteristics, compression-twisting properties, tunable mechanical properties, simultaneous stretching- and compression-expanding behaviors, negative static volume compressibility, and superior stiffness- and strength-to-weight ratios have all been designed and 3D printed thus far [9]. After external stressors are removed, multistable lattice structures can achieve many stable equilibrium states that are different from their initial undeformed condition. Common examples of bistable structures in nature are earwig wings, a mantis shrimp’s dactyl heels, and the leaf of the Venus flytrap. Furthermore, the design of novel functional structures and devices, such as deployable structures, mechanical logic gates, controllable structures, programmable structures, novel actuators, and energy absorbers and trappers, has shown a great deal of interest in artificial multistable lattice structures [10].

**Figure 1:**
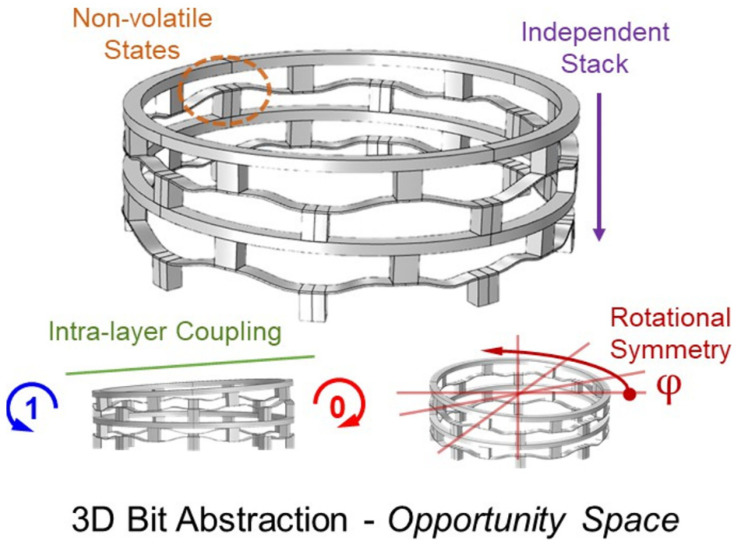
Exploring the 3D mechanologic opportunity space: innovations in bit abstractions with multistable axi-symmetric lattices. The proposed design features a latticed cylindrical structure composed of locally bistable unit cells. By coupling the kinematics of these bistable unit cells with a conductive polymer network, this design aims to achieve a high bit density through 3D, non-volatile abstractions.

Bistable materials are also used for structure building. In order to fulfill the worldwide targets for reduced carbon emissions, it is imperative that buildings increase their energy efficiency and decrease their carbon footprint. The emphasis on the building sector is appropriate: worldwide, the energy consumption of residential and commercial buildings has already surpassed other important sectors like industry and transportation, with the building sector accounting for approximately 40% of all primary energy consumption and related greenhouse gas emissions in the US [11]. Structure envelopes, which are the spaces in between the inside and outside, are largely responsible for a structure’s environmental performance. It is predicted that better building envelope design might result in a 40–60% reduction in the site’s overall energy usage. Moreover, the bulkiness and rigidity of many conventional electronics hinder the precise control and intricate movements required by autonomous robots [12,13]. However, recent advancements in manufacturing and materials science have paved the way for the creation of flexible, adaptive materials with integrated logic based on mechanical principles, offering a promising alternative to traditional electronic circuits. One innovative example of this progress is the development of digital logic within compliant materials. This has been accomplished by combining soft metamaterials with conductive polymer networks. The conductive polymer networks’ connections are manipulated through the buckling-driven kinematics of the metamaterial, as illustrated in Figure 1. In this configuration, the digital abstraction depends on volatile states restricted to one-dimensional deformation (left/right). Additionally, other alternatives to conventional electronics utilize bistable mechanisms to achieve non-volatile digital abstractions. For instance, the “waterbomb” origami design, shown in Figure 1 features two stable states and has been employed to create simple logic gates when paired with a humidity-responsive film that facilitates state transitions. This paper introduces a novel approach to bistable material design by integrating stiff and flexible components within a single unit cell, a method not extensively explored in the existing literature. The innovative combination leverages the structural integrity of hard materials with the energy absorption and elasticity of soft materials, resulting in a composite that transitions seamlessly between flexible and rigid states. This study employs advanced 3D printing techniques and comprehensive stress simulations to analyze the behaviors of these bistable composites under various conditions. Unlike traditional bistable materials, our approach demonstrates superior load distribution and stress dissipation capabilities, making it highly suitable for high-stress applications such as aerospace and automotive industries. By pioneering this unique integration and thoroughly examining its performance through both simulations and experimental validation, this paper significantly contributes to the advancement of bistable materials, offering new insights and potential for future applications.

## 2. Model Description

This model describes a novel composite material designed to achieve bistable properties through the strategic combination of soft and hard materials. The integration of these materials results in a composite that exhibits exceptional stiffness and versatility, making it suitable for a wide range of applications. In this research, we have studied both soft and hard materials. For the soft materials, we examined Thermoplastic Polyurethane (TPU), which is a highly elastic material with a stiffness (Young’s Modulus) ranging from 10 to 100 MPa. TPU can elongate up to 300–700% before breaking and has a tensile strength of 30–50 MPa. It typically does not have a distinct yield point, as it behaves elastically up to its breaking point. Another flexible material we studied is Thermoplastic Elastomer (TPE), which has a stiffness of 10–50 MPa and an impressive elongation at break of 400–1000%. TPE’s tensile strength ranges from 5 to 30 MPa, and like TPU, it generally lacks a clear yield point due to its elastic nature.

In addition to the soft materials studied, we have also examined several hard materials used in 3D printing, including Polylactic Acid (PLA), Acrylonitrile Butadiene Styrene (ABS), and Polyethylene Terephthalate Glycol (PETG). PLA is a biodegradable thermoplastic derived from renewable resources like cornstarch and sugarcane and is known for its rigidity, with a Young’s Modulus of about 2.7–16 GPa, elongation at break of up to 6%, and tensile strength ranging from 50 to 70 MPa. It is easy to print with and offers good detail resolution, though it is less durable and can become brittle over time. ABS, on the other hand, is celebrated for its toughness and impact resistance, featuring a Young’s Modulus of 1.1–2.7 GPa, elongation at break of 10–50%, and tensile strength of 30–55 MPa. ABS can withstand higher temperatures, making it suitable for functional parts, but requires careful handling due to its tendency to warp and emit fumes during printing. PETG bridges the gap between PLA and ABS by offering a combination of ease of use, strength, and durability. It has a Young’s Modulus of 2–2.3 GPa, impressive elongation at break of up to 300%, and tensile strength of 50–60 MPa. PETG is chemically resistant, moisture resistant, and prints with minimal warping, making it ideal for outdoor parts and food-safe containers. After examining many hard materials, we have chosen to use PLA, which can be combined with TPU for our cell unit and for testing. This combination leverages the rigidity and ease of printing of PLA with the flexibility and durability of TPU, providing a balanced approach to meet the specific requirements of our project.

In our experiment, we systematically applied forces using a Universal Testing Machine (UTM) to examine the effects of load compression and tension on the material. We began with an initial load of 1 Newton (N) and progressively increased it up to 80 Newtons. The UTM recorded a video of the test at intervals of 0.5 s, allowing us to closely observe the material’s response over time. As we applied increasing loads, both tensile and compressive stresses were introduced, enabling us to assess the material’s behavior under varying conditions. At lower loads, the material exhibited minimal deformation, while higher forces tested its ability to sustain and recover from both compressive and tensile stresses. This detailed video documentation provided valuable insights into the material’s performance throughout the testing process.

A mathematical model for the unit cell simulation was developed using COMSOL Multiphysics software, version 5.3. The model employs partial differential equations (PDEs) and specific boundary conditions to address mass balance and charge distribution within the unit cell. To ensure accurate results, a relative tolerance of 0.001 was applied, with solution convergence achieved within 30 to 45 iterations. The mesh for the simulation comprised approximately 884,835 tetrahedral elements, 243,543 triangular elements, 16,345 edge elements, and 1789 vertex elements. Spatial discretization was performed using the finite difference method (FDM).

**Figure 2:**
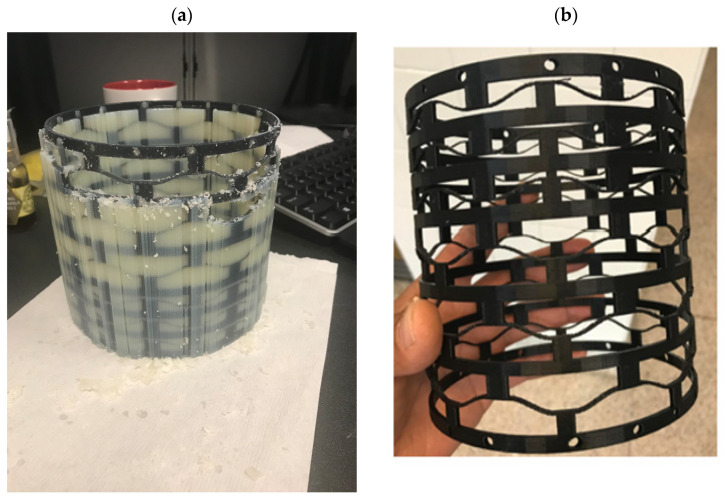
Comparison of 3D printing techniques and material properties for Thermoplastic Polyurethane (TPU) (**a**) and Polylactic Acid (PLA) (**b**). This figure contrasts the 3D printing processes and material characteristics of TPU and PLA. TPU, a flexible material, is printed with a nozzle temperature of 210–230 °C and a bed temperature of 40–60 °C. In contrast, PLA, a rigid material, is printed with a nozzle temperature of 180–220 °C and a bed temperature of 50–70 °C, resulting in more rigid and precise components.

The bistable behavior of the composite is achieved through the careful design of its internal structure, allowing the material to exist in two stable states. In Stable State 1, the composite exhibits high flexibility and can easily conform to different shapes, with the soft material dominating the mechanical behavior, enabling the effective absorption of shocks and vibrations. In Stable State 2, the composite transitions to a high-stiffness configuration, with the hard material predominating, providing exceptional rigidity and resistance to deformation under specific loading conditions. This transition allows the material to support heavy loads and maintain structural integrity, as shown in Figure 2.

The unique combination of soft and hard materials in a bistable configuration results in a composite with outstanding performance characteristics. Enhanced stiffness is achieved through the hard material component, making the composite suitable for structural applications where load-bearing capacity is critical. The versatility of the material, with its ability to switch between flexible and rigid states, allows it to adapt to various operational conditions, which is particularly beneficial in dynamic environments. The composite’s flexible state effectively absorbs energy from impacts and vibrations, protecting underlying structures and increasing its durability. Additionally, optimizing the proportion of soft and hard materials achieves a high strength-to-weight ratio, making the composite ideal for applications where weight reduction is essential.

The bistable composite material can be utilized in a wide array of applications, including in the automotive industry for components that need to absorb impact energy during collisions yet remain rigid under normal driving conditions, aerospace engineering for lightweight structures with high stiffness for load-bearing applications such as wing components and fuselage sections, robotics for flexible joints that need to transition to a rigid state to support heavy loads or precise movements, and protective gear in body armor and helmets where energy absorption during impacts is critical but stiffness is required for protection. The development of bistable composite materials combining soft and hard components represents a significant advancement in materials science, offering a unique blend of flexibility and rigidity, enabling high performance across diverse applications. The ability to tailor the material properties to specific needs opens new possibilities in design and engineering, paving the way for innovative solutions in various industries.

### One-Unit Cell Model Description

**Figure 3:**
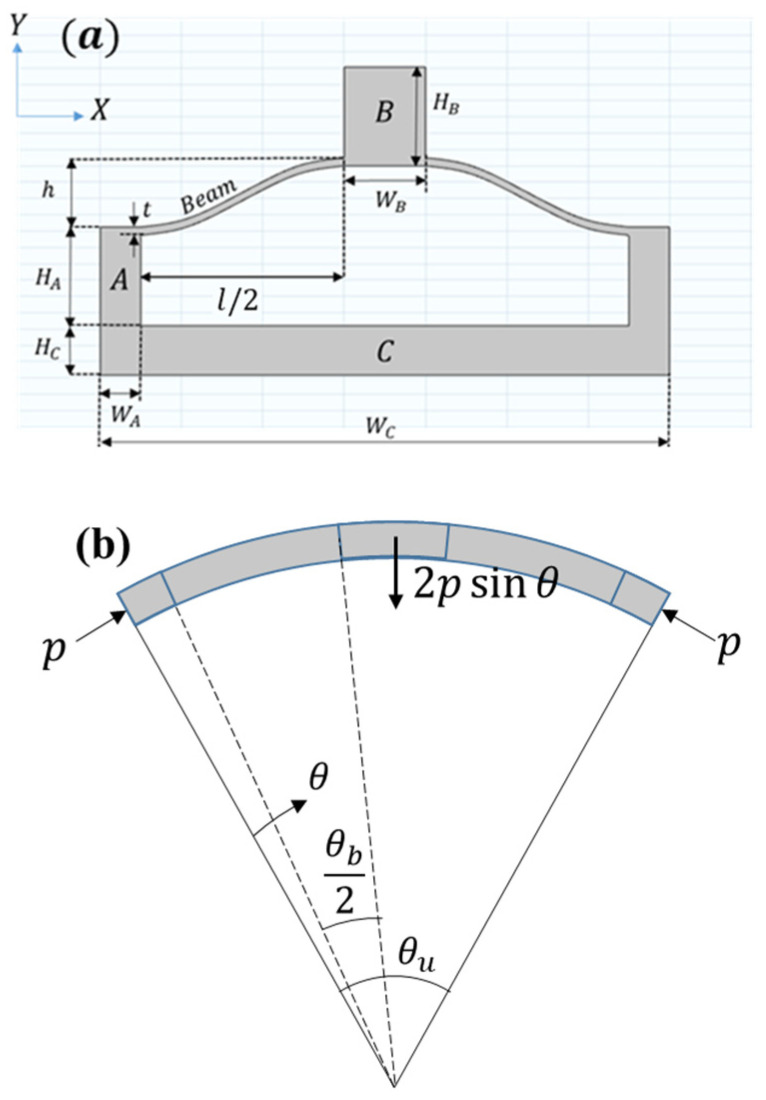
Schematic of a bistable unit consisting of three types of blocks, A, B, and C, and a pre-curved beam, is shown from a (**a**) side view. The curvature of the unit cell is shown from a top view in (**b**).

The pre-curved beam mechanism serves as the basis of the articulating multistable cylinder. As illustrated in Figure 3, the pre-curved beams are wrapped on a cylindrical surface with a polar angle $\theta_{b}$, with the entire unit cell covering $\theta_{u}$. The circumference of the cylinder can be equally divided by $\theta_{u}$ such that the total number of circumferential unit cells n is a positive integer defined by $n=2\pi /\theta_{u}$. Furthermore, it is assumed that n is an even number, which will be convenient in later derivations.

The Euler–Bernoulli beam equation is modified for a pre-curved beam warped on a cylinder,

(1) $$\frac{EI}{R^{4}}\frac{d^{4}w}{d\theta^{4}}+\frac{p\cos\left(\theta_{b}-\theta \right)}{R^{2}}\frac{d^{2}w}{d\theta^{2}}=0,$$

where w is the deflection of the beam as a function of θ, E is the elastic modulus, I is the moment of inertia, $\theta_{b}$ is the angle of the section in the polar coordinates, and p is the axial force in the beam, as shown in Figure 2. Following the derivations of [14], a constant pk can replace $p\cos\left(\theta_{b}-\theta \right)$ as the axial force acting on the beam through its centerline, normalized by

(2) $$N^{2}=\frac{pkL^{2}}{EI},$$

where N is the normalized axial force, and L is the arc length of the pre-curved beams in Figure 2a. For unit cells covering a sufficiently small section of the cylinder (i.e., $\theta_{b}\ll 2\pi$), the value of pk→p.

Given clamped–clamped boundary conditions, $w\left(0\right)=w\left(\theta_{b}\right)=0$, and ${\left(\frac{dw}{d\theta }\right)}_{\theta =0}={\left(\frac{dw}{d\theta }\right)}_{\theta =\theta_{b}}=0$, along with the relation in (4), the solution to (3) has the forms [15]

(3) $$\left.\begin{array}{c} w_{j}=C\left[1-\cos\left(N_{j}\frac{\theta }{2\pi /n}\right)\right]\\ N_{j}=(j+1)\pi \end{array}\right\},\;\;\;\;j=1,3,5,\ldots$$

and

(4) $$\left.\begin{array}{c} w_{j}=C\left[1-\frac{n\theta }{\pi }\right]-\cos\left(N_{j}\frac{\theta }{2\pi /n}\right)+\frac{2\sin\left(N_{j}\frac{n\theta }{2\pi }\right)}{N_{j}}\\ N_{j}=2.86\pi ,4.96\pi ,\ldots \end{array}\right\},\;\;\;\;j=2,4,6,\ldots$$

The solutions given in (3) and (4) are the foundation for the analytical modeling of the beams, as they constrain the system to a specific set of shapes. Furthermore, these forms correspond to buckling modes of order j. Prior studies have found that while higher-order buckling modes can provide more accurate results, the first three modes suffice for close approximations [14,15]. Typically, the second mode is constrained when applying a symmetric load, leaving just the first and third modes. For the bending cylinder analysis, it is justifiable to keep the second mode unconstrained, since the articulation will yield a slight beam rotation at the center of each unit cell, and thus, the typical boundary condition requiring a zero slope at the center of the beam, i.e., ${\left(\frac{dw}{d\theta }\right)}_{\theta =0}=0$, does not apply.

The primary geometric parameters of the unit cell are the height, horizontal length, and thickness of the beams, expressed as h, L, and t, respectively. The initial shape of the pre-curved beams as a planar structure is given by

(5) $$w_{0}=\frac{h}{2}w_{1},$$

where $w_{0}$ is the initial vertical height of the beam, $w_{1}$ is the first buckling mode, and

(6) $$w_{1}\left(x\right)=1-cos(N_{j}\frac{\theta }{2\pi /n}).$$

In addition to the bending energy, the beam will also experience compression that is related to the axial force p given by Hooke’s law,

(7) $$p=Ebt\frac{s-s_{0}}{s},$$

where s is the arc length of the beam, and $s_{0}$ is the initial arc length. The arc length is defined in polar coordinates as

(8) $$s=\int_{0}^{2\pi /A}\left[{\left(R^{2}+\frac{1}{2}{\left(\frac{dw}{d\theta }\right)}^{2}\right)}^{2}\right]d\theta .$$

Given (1), (7), and (8), the variations of the bending energy $u_{b}$, compressive energy $u_{c}$, and potential of the force $u_{f}$ can be written as

(9) $$\begin{array}{c} \partial \left(u_{b}\right)=\partial \left[\frac{EI}{2}\int_{0}^{\frac{\pi }{n}}{\left(\frac{d^{2}w_{0}}{d\theta^{2}}-\frac{d^{2}w}{d\theta^{2}}\right)}^{2}d\theta \right],\\ \partial \left(u_{c}\right)=p\partial \left(s\right),\\ \partial \left(u_{f}\right)=f\partial \left(d\right). \end{array}$$

To solve for the beam deflection, a superposition of the buckling mode shapes is employed. While other shapes could be used in superposition as well, the buckling modes more accurately represent the physics of the snap-through behavior. Furthermore, the literature has found that the first, second, and third modes play the most significant role in minimizing the energy of the beam [15]. Assuming that higher mode shapes are negligible, the solution is a linear combination

(10) $$w=A_{1}w_{1}+A_{i}w_{i},$$

in which $A_{1}$ and $A_{i}$ are coefficients serving as the primary parameters for the solution. If the second mode is constrained, then the coefficient $A_{i}$ is substituted with $A_{3}$; if the second mode is not constrained, then $A_{i}=A_{2}$, and consequently, $w_{i}=w_{2}$.

All variables present in the energies in (9) and the solution forms (3) and (4) can be normalized using the following relations,

(11) $$\begin{array}{ccc} F=\frac{fL^{3}}{EIh} & ∆=d/h & S=\frac{sL}{h^{2}}\\ N^{2}=\frac{peL^{2}}{EI} & U_{b}=\frac{u_{b}L^{3}}{EIh^{2}} & U_{c}=\frac{u_{c}L^{3}}{EIh^{2}}\\ U_{f}=\frac{u_{f}L^{3}}{EIh^{2}} & & \end{array}$$

Assuming the second mode is constrained, implementing the normalizations and summing $\partial \left(U_{i}\right)$ gives the variation in the total energy,

(12) $$\partial \left(U_{t}\right)=\left(\frac{N_{1}^{2}}{2}A_{1}-\frac{N_{1}^{2}}{2}-\frac{N^{2}N_{1}^{2}}{2}A_{1}K_{2}+2F\right)\partial \left(A_{1}\right)+\left(\frac{N_{3}^{2}-K_{2}N^{2}N_{3}^{2}}{4}\right)\partial \left(A_{3}^{2}\right).$$

Minimizing the energy yields two solutions depending on the value of $N^{2}$, which is determined by (13). If $N^{2}<N_{3}^{2}/K_{2}$, then $A_{3}=0$, leaving just the solution of the first kind,

(13) $$F_{1}=\frac{3\pi^{4}Q^{2}K_{2}}{2}∆\left(∆-\frac{3}{2}+\sqrt{\frac{1}{4}-\frac{4}{3K_{2}Q^{2}}}\right)\left(∆-\frac{3}{2}-\sqrt{\frac{1}{4}-\frac{4}{3K_{2}Q^{2}}}\right).$$

When $N^{2}=N_{3}^{2}/K_{2}$, then $A_{3}\neq 0$, yielding the solution of the second kind,

(14) $$F_{3}=\frac{1}{K_{1}}\left(8\pi^{4}-6\pi^{4}∆\right).$$

and likewise, for the third kind,

(15) $$F_{2}=2\pi^{4}\left[\left(N_{2}^{2}-N_{1}^{2}\right)\left(1-∆\right)+1\right],$$

## 3. Results and Discussion

**Figure 4:**
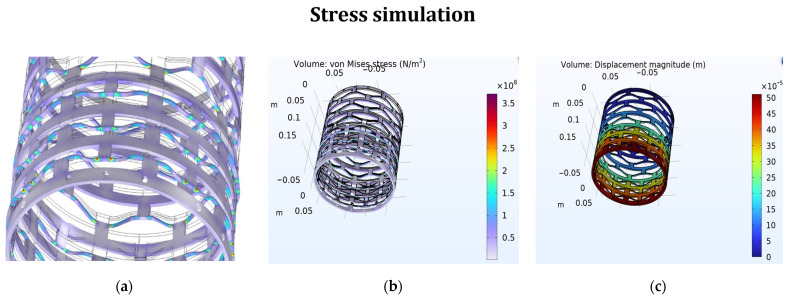
Stress simulation for the bistable model (**a**,**b**). Displacement of the shape (**c**).

The results reported in Figure 3 from the 3D simulations using COMSOL Multiphysics highlight several critical aspects of the behaviors of bistable materials under stress and acoustic pressure. The stress simulation results indicate that the bistable materials effectively absorb stress at the edges before it reaches the main body of the shape, providing an initial buffer that protects the core structure. This edge absorption is crucial for maintaining the integrity of the material under high-stress conditions. Moreover, the displacement analysis depicted in Figure 4 shows that the first few layers of the material’s body absorb the majority of the displacement. Specifically, the initial layers experience significant deformation, but by the time the stress wave reaches the fourth and fifth rows, the displacement declines dramatically. This rapid attenuation indicates that the material’s design effectively dissipates energy, preventing excessive movement from propagating through the structure. The simulations thus confirm that bistable materials not only absorb and localize stress at the edges but also efficiently reduce displacement within the main body, enhancing their suitability for applications requiring robust impact resistance and durability.

**Figure 5:**
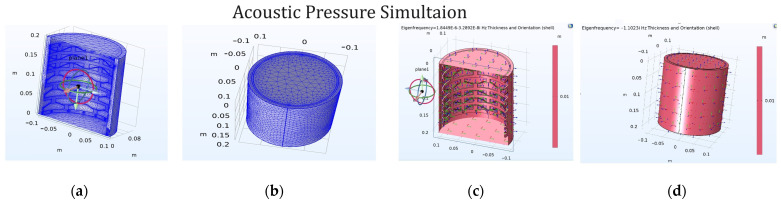
Acoustic pressure simulation (**a**,**b**). Shape fragment direction during high pressure (**c**,**d**).

In this study, we chose to perform COMSOL Multiphysics Acoustic simulations on hard materials because the soft materials could not adequately withstand the parameters of the study. Figure 5 presents the results of acoustic pressure simulations for a bistable shape under extreme pressure conditions. The simulations illustrate that when pressure is applied in an attempt to expand a tank made of the bistable shape, the first three layers of the material absorb the majority of the pressure, as depicted in Figure 4a,b. This initial absorption prevents the pressure from propagating further into the tank, thereby protecting the core structure. Additionally, Figure 4c demonstrate the behavior of the bistable shape when configured into a cylindrical form. Under pressure, the cylinder fragments and the pressure spread in all directions—x, y, and z—indicating a uniform dispersion of force. This directional spread of pressure highlights the material’s potential to manage and dissipate energy efficiently. These characteristics are particularly advantageous for applications in the aerospace and military industries, where materials are required to withstand high pressures and impacts while maintaining structural integrity. The bistable shape’s ability to absorb and disperse pressure effectively makes it an ideal candidate for protective structures and components in these fields.

**Figure 6:**
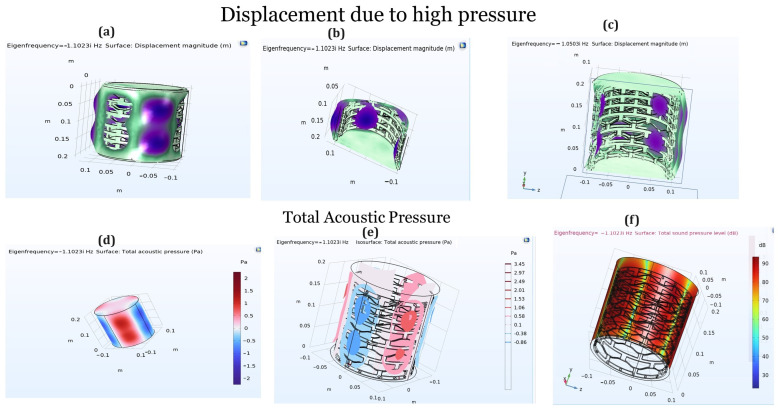
Displacement magnitude of the bistable shape (**a**–**c**) due to high pressure and (**d**–**f**) total acoustic pressure.

Figure 6 presents the behavior of a bistable shape configured into a cylindrical form under extremely high pressure. The simulation results, as shown in Figure 6a–c, indicate that one side of the cylinder undergoes significant expansion, while the adjacent column exhibits less expansion. This alternating pattern of high expansion followed by low expansion is due to the bistable shape’s unique ability to absorb and dissipate energy efficiently. The bistable design creates a controlled deformation pattern, allowing the material to manage and distribute the applied pressure more effectively across the structure, thereby preventing catastrophic failure. Additionally, Figure 6e,f show acoustic pressure simulations for different column views. These figures demonstrate how the columns of the bistable shape behave under pressure compared to the previous figures, which focus on different rows. The columnar behavior observed in these simulations aligns with the earlier findings, highlighting the material’s capacity to absorb and dissipate energy uniformly. This consistent performance across various orientations is extremely promising for numerous applications, particularly in fields such as aerospace and military industries, where materials must withstand and mitigate high-pressure impacts. The results confirm the bistable shape’s potential for enhancing the durability and resilience of structures exposed to extreme conditions.

**Figure 7:**
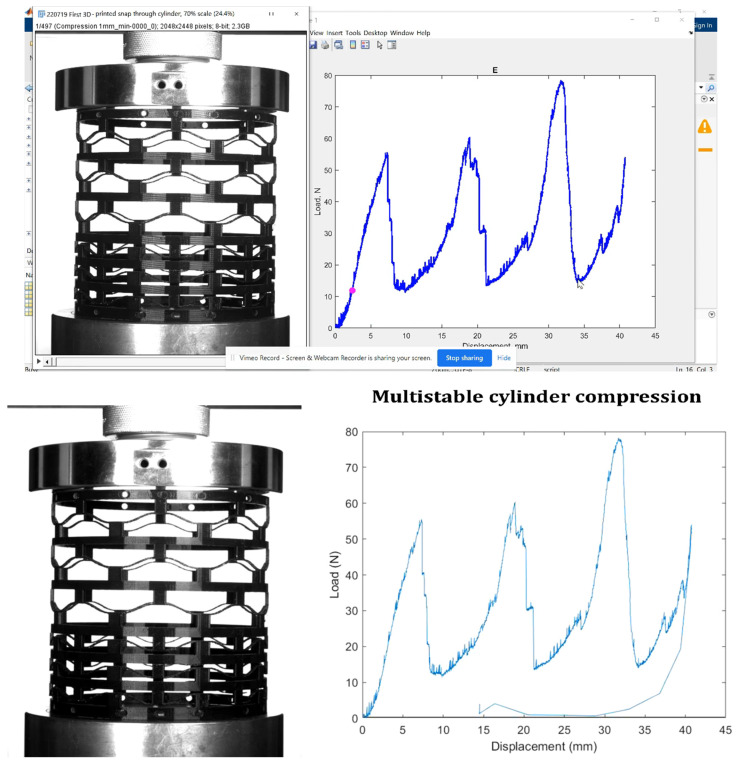
Compression test on a 3D-printed ABS material of the cylindrical bistable shape.

Figure 7 illustrates the results of a compression test conducted on a 3D-printed ABS material of a cylindrical bistable shape. The test demonstrates that each layer of the cylindrical structure can hold a significant load before failing. As the load increases, the stress is initially absorbed by the first layer, causing the curve to rise. Once the bistable unit cells in the first layer start to break, the load is transferred to the next layer, resulting in a sudden drop in the curve. This pattern repeats as each subsequent layer absorbs the load and then breaks, leading to a series of rises and drops in the curve. This sequential load-bearing and failure mechanism showcases the material’s ability to manage and distribute stress efficiently across multiple layers. The results from this compression test indicate that the bistable cylindrical shape has excellent energy absorption and load distribution properties, making it highly suitable for aerospace applications. In aerospace structures, materials must endure high stress and impact while maintaining structural integrity. The bistable design’s ability to progressively absorb and distribute the load through multiple layers enhances the durability and resilience of aerospace components, providing a robust solution for environments where mechanical reliability and energy dissipation are critical.

**Figure 8:**
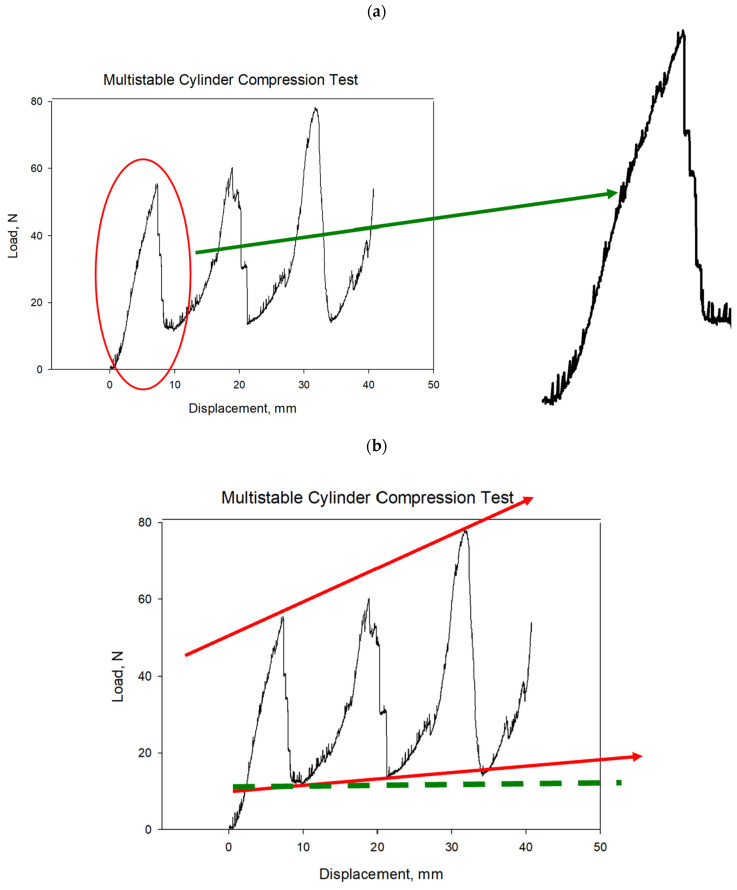
Multistable compression test vs. displacement of the cylindrical bistable shape (**a**,**b**) (Red line: Multistable load trend; Green line: Snap-off cell row load).

Figure 8 illustrates the multistable compression test results versus displacement for the cylindrical bistable shape. Each line in the graph represents the behavior of a single unit cell within the structure under compressive load. As the test progresses, individual unit cells begin to break, causing small, zigzag patterns in the line as the load fluctuates slightly up and down. This zigzagging continues until the entire first layer of unit cells has failed. Once the first layer is completely broken, the load is transferred to the next layer, which can bear a heavier load than the previous layer. In the second layer, the unit cells start to break in a similar manner, creating another series of zigzags in the line. This pattern repeats, with each layer absorbing more load than the previous one before breaking. When a layer fails completely, there is a noticeable drop in the load line, but each successive drop occurs at a higher load level, indicating that each new layer can bear more weight than the one before. This sequential failure and load transfer mechanism demonstrates the material’s capacity to manage stress and maintain structural integrity under increasing loads, highlighting its potential applications in the aerospace industry, where materials must reliably withstand high pressures and impacts.

**Figure 9:**
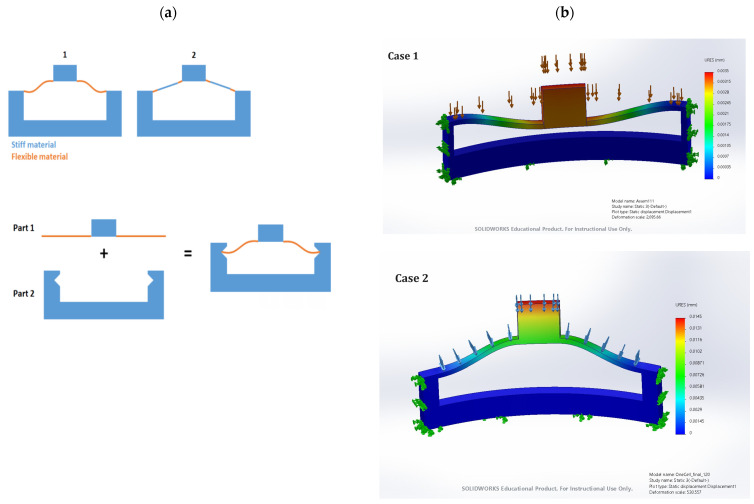
(**a**) New proposal of bistable materials with combination of stiff materials and flexible materials. (**b**) combined bistable materials deflection simulation.

In Figure 9, we provide a detailed explanation of the basic functioning of a single unit within the bistable structures to offer a clearer understanding of the mechanism behind bistability. The bistability arises from the interplay between the soft and stiff components, where the soft component allows for significant deformation, while the stiff component provides the necessary restoring force. This combination results in a structure capable of maintaining two stable configurations, offering unique advantages in terms of energy absorption, load distribution, and adaptability. By understanding the behavior of a single unit, the benefits of incorporating such bistable structures into larger systems become more apparent, as they contribute to enhanced performance and resilience.

The new proposal for bistable materials involves combining stiff and flexible materials within a single unit cell to observe potential changes in behavior. Figure 8 illustrates this concept, suggesting that the integration of these two materials within a bistable unit cell could enhance its performance. Initial stress simulations indicate promising bistable behavior, with stress levels significantly declining as they move away from the initial load area. This suggests that the combination of stiff and flexible materials can effectively manage and dissipate stress, maintaining structural integrity. Experimental results will follow to validate these findings, examining how the combined bistable material responds to compression tests. These tests will determine if the integration of stiff and flexible materials improves the overall performance, offering enhanced energy absorption and load distribution capabilities.

**Figure 10:**
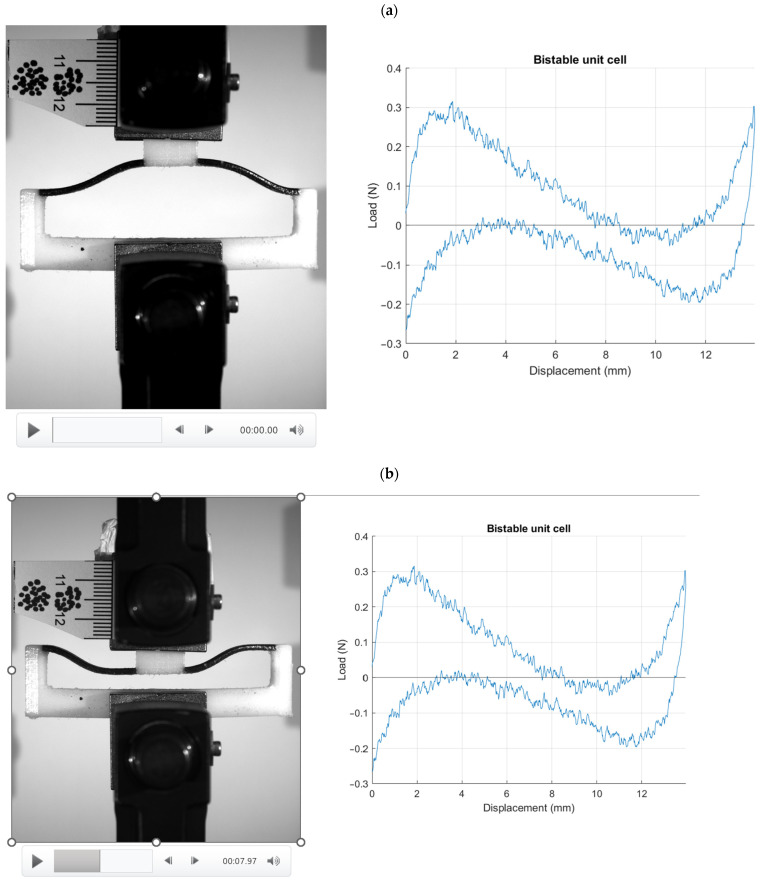

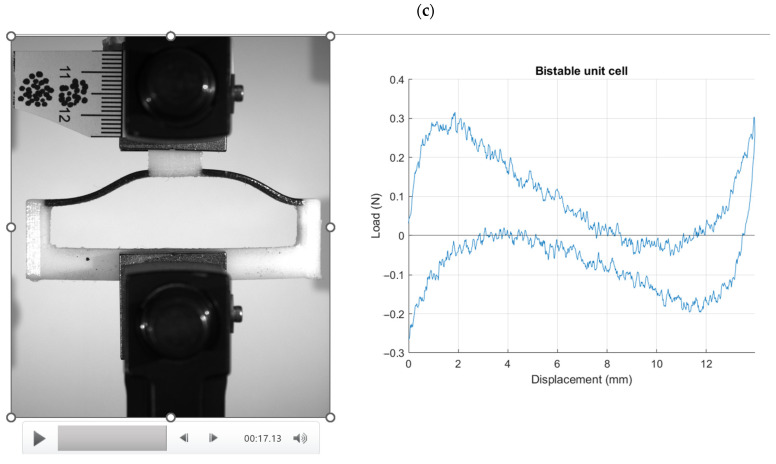
Video recordings of stiff and flexible unit cell under compression test: (**a**) beginning, (**b**) during, at 7 s, and (**c**) at 17 s of the compression test.

Figure 10 illustrates the results from the compression test on a unit cell combining stiff and flexible materials, captured in three distinct phases: (a) the beginning, (b) during, at 7 s, and (c) at 17 s of the compression test. The recorded video shows the unit cell’s performance under compressive load, highlighting its bistable behavior. The combination of stiff and flexible materials within a single unit cell exhibits excellent resilience, consistently returning to its initial shape after the load is removed. During the initial phase (Figure 10a), the unit cell is shown before any load is applied, illustrating its baseline configuration. At the 7 s mark (Figure 10b), the unit cell undergoes significant deformation due to the applied compressive force, demonstrating how the flexible components absorb energy and allow for temporary deformation. By 17 s (Figure 10c), the unit cell has experienced the full extent of the compressive load and has begun to return to its original shape upon load removal.

Combining stiff and flexible materials within a single unit cell has resulted in excellent bistable behavior, significantly enhancing the material’s performance under load conditions. When subjected to compressive stress, the combined unit cell exhibits a remarkable ability to return to its initial shape after the load is removed. This resilience is attributed to the synergistic effect of the stiff materials providing structural integrity and the flexible materials offering elasticity and energy dissipation. The unit cell maintains consistent load trends, showing nearly identical patterns during the loading and unloading phases. This behavior indicates that the unit cell can effectively absorb and release energy without permanent deformation, which is a hallmark of bistable materials. The stress distribution within the unit cell shows that it can handle significant loads while retaining its structural integrity, and once the load is removed, the unit cell’s original shape is restored. This predictable and repeatable performance under cyclic loading conditions is highly beneficial for applications requiring durability and reliability. The ability of the combined unit cell to maintain its shape and load-bearing capacity makes it suitable for various designs and applications, particularly in fields that demand high resilience and energy absorption, such as the aerospace, automotive, and protective gear industries. The results suggest that incorporating such bistable unit cells into different structures could lead to materials and components that are not only stronger and more durable but also capable of withstanding repetitive loading without degradation. This innovative approach to material design holds great promise for developing advanced materials with superior performance characteristics that are suitable for a wide range of engineering and industrial applications [16,17,18].

In our study, the mechanical loading was carefully controlled by applying a displacement-driven approach, where the displacement served as the independent variable, and the resulting load was the dependent variable. This method allowed us to precisely manipulate the displacement and accurately capture the corresponding load values, providing robust data for our analysis. The use of displacement-controlled loading is crucial for understanding the mechanical behavior under predefined deformations, offering insights into the material’s response and stability. A detailed explanation of this methodology, including its significance and application within our experiments, has been thoroughly discussed in the manuscript. This approach ensures that our results are both reliable and reproducible, contributing valuable data to the field. The unit cell of bistable structures offers significant advantages due to its unique mechanical behavior. Each unit cell is designed to exhibit bistability, meaning it can hold two distinct stable configurations. This characteristic allows for the efficient storage and release of mechanical energy, providing superior performance in applications requiring controlled actuation and movement. In aerospace engineering, such bistable unit cells can be particularly valuable for their lightweight and compact nature, which is essential for optimizing the payload capacity and enhancing the functionality of deployable structures and mechanisms. By leveraging the bistable properties of these unit cells, aerospace systems can achieve greater efficiency and reliability in various applications, including deployable antennas, structural components, and adaptive wing structures.

## 4. Conclusions

This study underscores the significant potential of bistable structures and materials in advancing the design and functionality of aero-vehicles and other high-stress applications. The findings demonstrate how bistable materials, particularly those integrating stiff and flexible components, exhibit superior performance under various stress conditions while maintaining structural integrity and resilience. The simulations and experiments discussed in this study reveal that bistable materials effectively absorb and dissipate stress, particularly at the edges and initial layers, thereby protecting the core structure. This behavior is advantageous for applications requiring materials to withstand high pressures and impacts while maintaining durability. The compression tests on 3D-printed ABS cylindrical bistable shapes showed that these materials could bear significant loads, with the stress distribution managed efficiently across multiple layers. The observed sequential failure and load transfer mechanism highlight the material’s capability to sustain and distribute stress, making it highly suitable for aerospace applications where mechanical reliability and energy dissipation are critical. Moreover, the proposal to combine stiff and flexible materials within a single unit cell represents a novel concept that has not been extensively explored in the literature. The initial stress simulations and compression test results confirmed that these combined materials return to their initial shape after load removal, maintaining consistent load-bearing patterns. This characteristic is particularly beneficial for applications that require repeated loading cycles without permanent deformation, such as in aerospace, automotive, and protective gear sectors. The innovative use of bistable materials, especially those integrating both stiff and flexible components, offers a promising avenue for developing advanced materials with exceptional performance characteristics. These materials can provide enhanced durability, energy absorption, and resilience, making them ideal for a wide range of engineering and industrial applications. The findings of this study pave the way for further exploration and optimization of bistable structures, potentially revolutionizing material design and application in high-stress environments while contributing new insights to the open literature.

## Data Availability

Data is unavailable due to privacy.
